# Effect of age on pro-inflammatory miRNAs contained in mesenchymal stem cell-derived extracellular vesicles

**DOI:** 10.1038/srep43923

**Published:** 2017-03-06

**Authors:** J. Fafián-Labora, I. Lesende-Rodriguez, P. Fernández-Pernas, S. Sangiao-Alvarellos, L. Monserrat, O. J. Arntz, F. J. Van de Loo, J. Mateos, M. C. Arufe

**Affiliations:** 1 Grupo de Terapia Celular y Medicina Regenerativa (TCMR-CHUAC). CIBER-BBN/ISCIII. Servicio de Reumatología, Instituto de Investigación Biomédica de A Coruña (INIBIC), Complexo Hospitalario Universitario de A Coruña (CHUAC), SERGAS, Departamento de Medicina, Facultade de Oza, Universidade de A Coruña (UDC), As Xubias, 15006, A Coruña, Spain; 2 Grupo Fisiopatología Endocrina, Nutricional y Médica (FENM-CHUAC), Instituto de Investigación Biomédica de A Coruña (INIBIC), Complexo Hospitalario Universitario de A Coruña (CHUAC), SERGAS, Departamento de Medicina, Facultade de Oza, Universidade de A Coruña (UDC), As Xubias, 15006, A Coruña, Spain; 3 Cardiology Department, Health in Code, As Xubias, 15006, A Coruña, Spain; 4 Experimental Rheumatology, Radboudumc University Medical Center, Huispost 272, route 272, Postbus 9101, 6500 HB Nijmegen, The Netherlands

## Abstract

Stem cells possess significant age-dependent differences in their immune-response profile. These differences were analysed by Next-Generation Sequencing of six age groups from bone marrow mesenchymal stem cells. A total of 9,628 genes presenting differential expression between age groups were grouped into metabolic pathways. We focused our research on young, pre-pubertal and adult groups, which presented the highest amount of differentially expressed genes related to inflammation mediated by chemokine and cytokine signalling pathways compared with the newborn group, which was used as a control. Extracellular vesicles extracted from each group were characterized by nanoparticle tracking and flow cytometry analysis, and several micro-RNAs were verified by quantitative real-time polymerase chain reaction because of their relationship with the pathway of interest. Since miR-21-5p showed the highest statistically significant expression in extracellular vesicles from mesenchymal stem cells of the pre-pubertal group, we conducted a functional experiment inhibiting its expression and investigating the modulation of Toll-Like Receptor 4 and their link to damage-associated molecular patterns. Together, these results indicate for the first time that mesenchymal stem cell-derived extracellular vesicles have significant age-dependent differences in their immune profiles.

The promising role of mesenchymal stem cells (MSCs), whose mechanism of action is predominantly paracrine, in cell-based therapies and tissue engineering appears to be limited due to a declination of their regenerative potential with increasing donor age^1^. Next Generation Sequencing (NGS) is a versatile technology that allows the cataloguing of cellular constituents at a steady state and functional interactions when combined with system perturbation and differential analysis^2^. Together with novel methods of pattern recognition and network analyses^3^, NGS has revolutionized the field of Systems Biology. Samples from newborn, infant, young, pre-pubertal, pubertal and adult bone marrow-derived MSCs have been studied by NGS to evaluate the modifications of gene expression during ageing. Recently, the role of micro-RNAs in ageing and immunosenescence has been reported and their relevance to extracellular vesicles from MSCs affecting their therapeutic potential. Extracellular vesicles (EVs), such as exosomes or micro-vesicles, are released by cells into the environment as sub-micrometre particles enclosed by a phospholipid bilayer^4^. EVs have been found to mediate interactions between cells, mediate non-classical protein secretion, facilitate processes such as antigen presentation, participate in trans signalling to neighbouring cells and in the transfer of RNAs and proteins^5^. The detection of low copy numbers of mRNA and small RNAs, including micro-RNAs (miRNA), in EVs from mouse and human mast cell lines (MC/9 and HMC-1, respectively) has added much research interest impetus to the field^6^. While mRNA and miRNA in EVs are inactive, they have the potential to be active when EVs are transfected into nearby cells. Studies indicate that the EV miRNA expression profile may be of diagnostic/therapeutic potential^7^. The Toll-like receptors (TLRs), an important component of innate and adaptive immune responses^8^, are expressed in MSCs and their derived EVs during ageing. TLR4 signalling contributes to response specificity, leading to increased transcription of NF-κB and AP-1 target genes like IL-8, IL-6, IL-1β, TNF-α, and IFN-β^9^. Damage-associated molecular patterns (DAMPs) are molecules that have a physiological role inside but acquire additional functions when exposed to the extracellular environment, and they can be secreted or displayed by living cells undergoing a life-threatening stress^10^. Thus, we studied the changes in activation of Toll-Like receptor 4 (TLR4) together with expression changes in the DAMP S100 proteins including S-100A4, S100A6 and HMGB1, and their relationship with miRNA21-5p in pre-pubertal MSCs.

## Materials and Methods

### Isolation and culture of cells

For isolation of MSCs, the animals were anesthetized with Fluorane (Izasa, A Coruña, SP) and euthanized by cervical dislocation. Femurs from four male Wistar rats were dissected (Animal Service, CHUAC) at different ages: newborn (0 days old), infant (7 days old), young (14 days old), pre-pubertal (35–38 days old), pubertal (45 days old) and adult (108 days old). All the methods were carried out in “accordance” with the approved guidelines of Spanish law (32/2007). All experimental protocols were approved by the Animal Ethical Committee of Galicia. The protocol used by Karaoz *et al*.^11^ was followed in this work. Briefly, the ends of the bones were cut away and a 21-gauge needle that was inserted into 5h3 shaft of the bone marrow was extruded by flushing with 5 ml D-Hank’s solution supplemented with 100 IU/ml penicillin–1 mg/ml streptomycin (all from Life Technologies, Madrid, Spain). The marrow plug suspension was dispersed by pipetting it up and down, successively filtered through a 70-μm mesh nylon filter (BD Biosciences, Bedford, MA, USA), and centrifuged at 20,000 g for 10 min. The supernatant containing the platelets and erythrocytes was discarded, and the cell pellet was resuspended in the medium. The cells from four animals were seeded into 100 cm^2^ dish plates (TM Nunclon) and incubated at 37 °C with 5% humidified CO_2_. The MSCs were isolated based on their ability to adhere to the culture plates. On the third day, red blood cells and other non-adherent cells were removed by a pre-plating technique, and fresh medium was for further growth. The adherent cells grown to 80% confluence were defined as passage zero (P0) cells. After 5 min of centrifugation, 1 × 10^6^ MSCs were seeded on two 100 cm^2^ dish plates (TM Nunclon) in RPMI supplemented with 10% foetal bovine serum (FBS), 100 U/ml penicillin and 1 mg/ml streptomycin (all from Life Technologies, Madrid, SP). The medium was added and replaced every 3 or 4 days. MSCs were expanded for two passages and characterized by flow cytometry.

### RNA-Seq protocol

The study was designed to screen the complete transcriptome sequence per age group of Wistar rats. Sample preparation was carried out as recommended by Agilent SureSelect Strand-Specific RNA Library Prep for Illumina multiplexed sequencing method^12^. 1 μg of total RNA per sample was generated. The Sequencing data was generated on Hiseq 1500 on a rapid mode flow cell from Illumina. Sample preparation and sequencing was conducted in duplicate.

### Real-time quantitative polymerase chain reaction (qRT-PCR) analysis

RNA was isolated using the TRIzol® extraction method. The quality of 1 μL of each RNA sample was checked using the Agilent Bioanalyzer 2100 to determine the RIN (RNA Integrity) score using the Agilent 6000 Nanochip and reagents (Agilent, St. Clara, USA). Samples with a RIN score >7 were retained and converted to cDNA by SureSelect Strand Specific RNA library (Agilent, St. Clara, USA).

For miRNA detection, cDNA was generated from DNaseI-treated RNA, using a QuantiMir RT Kit (System Biosciences, CA, USA) according to the manufacturer’s instructions. PCR products were amplified using specific primers for miRNAs: rno-miR-335 (MIMAT0000575; 5′-TCAAGAGCAATAACGAAAAATGT); rno-miR-155-5p (MIMAT0030409; 5′-TTAATGCTAATTGTGATAGGGGT); hsa-miR-132-5p (MIMAT0004594, 5¨-ACCGTGGCTTTCGATTGTTACT); hsa-miR-146a (MIMAT0000449, 5¨-TGAGAACTGAATTCCATGGGTT); rno-miR-21-5p (MIMAT0000790, 5′-TAGCTTATCAGACTGATGTTGA) and hsa-miR-16 (MIMAT0000069, 5′-TAGCAGCACGTAAATATTGGCG). The amplification programme consisted of an initial denaturation at 50 °C for 2 minutes followed by 95 °C for 10 minutes and 50 cycles annealing at 95 °C, depending on the gene, for 15 seconds and extension at 60 °C for 1 minute. Primers for the amplification of rat genes are described in detail in Table 1. The amplification programme consisted of an initial denaturation at 92 °C for 2 minutes followed by 40 cycles from 92 °C for 15 seconds, annealing at 55–62 °C, depending on the gene, for 30 seconds and extension at 72 °C for 15 seconds. PCR analysis was done in triplicate, with each set of assays repeated three times. To minimize the effects of unequal quantities of starting RNA and to eliminate potential sources of inconsistency, relative expression levels of each gene were normalized to ribosomal protein (HPRT) or miR-16 using the 2^−ΔΔ^ Ct method^13^. Control experiments utilized no reverse transcriptase.

### Isolation extracellular vesicles

Bone marrow mesenchymal stem cells from newborn (0 days), young (14 days), pre-pubertal (35–38 days) and adult (3 months) were cultured in RPMI 1640 Medium with GlutaMAXTM supplement and 10% exosome-free FBS (all Thermo Fisher Scientific, Massachusetts, USA), 100 U/ml penicillin and 1 mg/ml streptomycin (Sigma-Aldrich, St. Louis, USA). Cells were cultured until 80% confluence and the supernatants were collected after 48 hours. We isolated MSC-derived EVs using ultracentrifugation following the protocol published by Del Fattore *et al*.^14^. In detail, supernatants were centrifuged at 1,500 rpm for 10 min at 4 °C and filtered using a sterile 0.22-μm filter (GE Healthcare Life Sciences, Maidstone, UK) to eliminate debris. Supernatants were transferred to ultracentrifugation tubes and centrifuged at 100,000 g for 2 hours at 4 °C in an Optimal-90K centrifuge with a 60 Ti rotor (Beckman Coulter, Mississauga, Canada). The supernatants containing exosome-free media were removed, and the pellets were resuspended in 200 μl PBS.

### Nanoparticle tracking analysis

The Brownian motion of the particles in a NanoSight LM12 using Nanoparticle Tracking Analysis 2.3 software (NanoSight Ltd., Amesbury, UK) was used to estimate the eVs size distribution. EVs were diluted in PBS until a suitable concentration for analysis was reached. Particle concentration was evaluated for particles ranging between 30–150 nm in diameter.

### Electronic microscopy

EVs were concentrated using Vivaspin concentrators (Sartorius, Gottingen, Germany). EVs were taken up in small volumes of deionized water, which were placed on nickel grids and allowed to dry for 45 minutes at 37 °C. The grids with EVs were then washed by transferring them onto several drops of deionized water. Negative contrast staining was performed by incubating the grids on top of drops of 6% uranyl acetate. Excess fluid was removed, and the grids were allowed to dry before examination on a Jeol JEM1400 Transmission electron microscope (Jeol, Tokyo, Japan).

### Flow cytometry

To characterize the different populations of MSCs from chronologically different animals, their MSCs were washed twice in PBS, then pre-blocked with 2% rat serum in PBS. The following direct antibodies were used: PE-conjugated mouse anti-rat CD34 (1:20 from DakoCytomation, Barcelona, SP); FITC-conjugated mouse anti-rat CD45 (1:20 BD Pharmingen, New Jersey, USA); PE-Cy5.5-conjugated mouse anti-rat CD90 (1:20 Immunostep, Salamanca, SP) and APC-conjugated mouse anti-rat CD29 (1:20 Immunostep, Salamanca, SP). The cells were washed with PBS after one hour of incubation with the corresponding antibody at room temperature. The stained cells were then washed twice with PBS, and 2 × 10^5^ cells were analysed with a FACSAria flow cytometer (BD Science, Madrid, SP). FACS data were generated by DIVA software (BD Science). Negative control staining was performed using FITC-conjugated mouse IgG1K isotype, PE-conjugated mouse IgG1K isotype, PE-Cy5.5-conjugated mouse IgG1K isotype and APC-conjugated mouse IgG1K isotype (all from BD Pharmingen).

### miRNA transitory transfections

MSCs were incubated with 40 nM hsa-miR-21-5p miRVana™ miRNA inhibitor or 40 nM control negative miRVana™ miRNA Mimic using the expression system following the manufacturer’s instructions. Validation by RT-PCR was done using Taqman®MicroRNA Assays following commercial instructions (all from Ambion, Applied Biosystems, Madrid, SP).

### Protein isolation and immunoblot analysis

The protein content into EVs was measured with a Micro-BCA kit (Thermo Scientific, Pierce, Rockford, USA) following the manufacturer’s instructions. Immunoblot analysis was performed on 40 μg of total protein extracted from MSCs, as previously described^15^. The blots were probed with antibodies directed against: LMNA/C (Acrix); Wnt5a (Acrix); TLR4 (Immnunostep); mTOR (Cell Signalling); HMGB1 (Abcam); pAKT; AKT and tubulin (all from Cell Signalling) or **β**-actin (Sigma-Aldrich) were housekeeping proteins used as loading controls. Secondary anti-rabbit (Cell Signalling) or anti-mouse (DAKO) antibodies were used to visualize proteins using an Amersham ECL Western Blotting Analysis System (GE Healthcare, Amersham Biotechnology, Manchester, UK). Ideal concentrations for each antibody were empirically determined. Working concentrations were 1:1000 of the recommended stock solutions.

### Bioinformatics analysis

An average of 25 million paired-end 100-bp reads was obtained per sample. The raw RNA-Seq reads for each sample were aligned to the reference rat genome browser (rn6 assembly) using Bowtie2 (bowtie-bio.sourceforge.net/index.shtml/) and Tophat2 (http://tophat.cbcb.umd.edu/). After alignment, raw sequence read depths were converted to estimate transcript abundance measured as fragments per kilobase of exons per million (FPKM), and the Cufflinks (http://cufflinks.cbcb.umd.edu/) of differentially expressed genes and transcripts were calculated with Cuffdidd. Each group was compared with a previous age group. The fold-change thresholds had to be greater than 1.2 and lower than 0.8. Identified genes with statistically significant changes were categorized according to their function, biological process and cellular component, using the R/Bioconductor package RamiGO (http://bioconductor.org/packages/release/bioc/html/RamiGO.html)^16^.

MicroRNA.org (http://www.microrna.org) was used as a resource of microRNA target predictions and expression profiles. Target predictions were based on the development of the miRanda algorithm^17^ and TargetScan^18^.

### Statistical analysis

All experiments were conducted in triplicate, and one representative is shown. Statistical non-parametric analysis (Mann-Whitney U and Kruskal-Wallis tests) was performed using GraphPad Prism6 (GraphPad Software, La Jolla, CA). Each group was compared with a previous group. A *p* value of less than 0.05 or 0.01 was considered statistically significant. All of the data are presented as the standard error of the mean.

## Results

**Figure 1:**
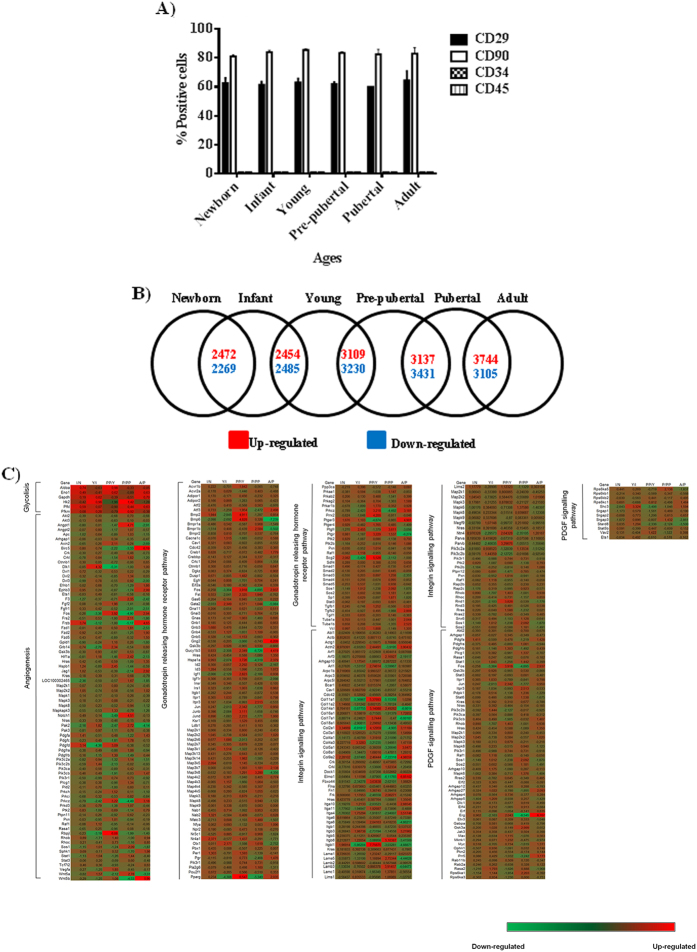
Characterization of mesenchymal stem cells. (**A**) Mesenchymal stem cell marker (CD29, CD90) and haematopoietic marker (CD34, CD45) signals were measured by flow cytometry. A representative flow graph for newborn group was shown in supplementary information. (**B**) Modified gene expression between age groups obtained in the RNA-Seq analysis. (**C**) Hierarchical clustering of genes from MSC age groups classified into metabolic pathways common to all of them.

The characterization of populations of MSCs from different age groups by flow cytometry indicated that these populations contained less than 1% of cells positive for CD45 and CD34 hematopoietic markers, more than 60 ± 5% cells positive for CD29 and more than 85 ± 5% cells positive for CD90 (Fig. 1A and Supplementary Information).

**Figure 2:**
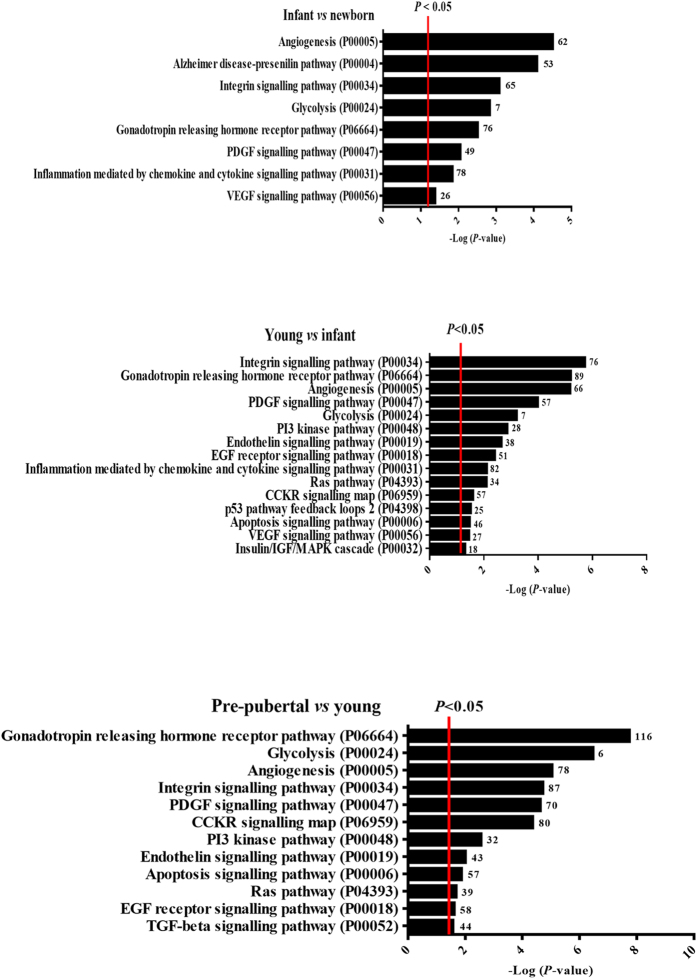
NGS study. Metabolic pathways with statistically significant changes between newborn, infant, young, pubertal and pre-pubertal age groups categorized according to their function, biological process and cellular component. Age groups were shown because of increasing differential gene expression involved in inflammation mediated by chemokine and cytokine signalling pathways. No genes involved in this pathway were identified between young and pre-pubertal age groups and between pubertal and adult age groups. Small numbers on the right of each bar are the modulated genes involved in each process.

**Figure 3:**
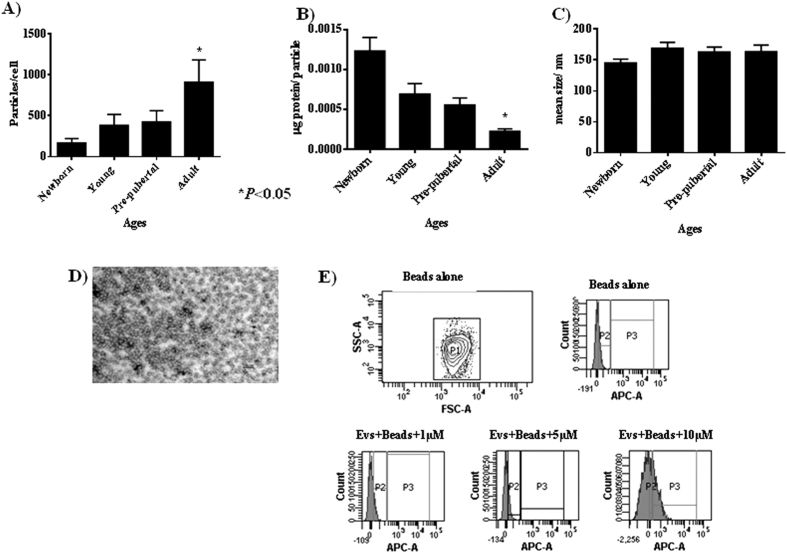
Characterization of mesenchymal stem cell-derived extracellular vesicles. (**A**) Number of particles per cell at different age groups by the NTA assay. (**B**) Concentration of protein per cell at different age groups by the NTA assay. (**C**) Mean size of particles expressed in nm at different age groups by the NTA assay. (**D**) Extracellular vesicles isolated from MSCs of the pre-pubertal group by microscopy electronic (scale bar = 100 nm). (**E**) APC-CD63 antibody signal measured by flow cytometry at different amounts (1, 5 and 10 μM) from pre-pubertal MSC-derived extracellular vesicles using beads.

NGS analysis indicated that 9,628 genes were differentially expressed between the selected age groups (Fig. 1B). Modulated genes are indicated as upregulated in red and downregulated in blue, chronologically and continuously comparing the age groups. The results indicated that the expression pattern of 4,741 genes change between newborn and infant groups; 4,939 genes change their expression pattern between infant and young groups; 6,339 genes change their expression pattern between young and pre-pubertal groups; 6,568 genes change their expression pattern between pre-pubertal and pubertal groups and 6,849 genes change their expression pattern between pubertal and adult groups. Figure 1C shows the hierarchical clustering of genes involved in five pathways common between the six age groups studied, using the R/Bioconductor package RamiGO with a signification of >1.5-fold. Genes modulated between newborn and infant groups were grouped into eight metabolic pathways, while genes modulated from infant until adult groups were grouped into up to 15 metabolic pathways. The number of modulated genes involved in hormonal changes including the gonadotropin-releasing hormone pathway (PO6664) increased from the infant age group until the adult age group (76, 89, 116, 112 and 121 genes, respectively). Genes involved in programmed death in the apoptosis signalling pathway (PO00006) were modulated between the young, pre-pubertal and adult groups (46, 57, 66, respectively). Genes involved in inflammation mediated by the chemokine and cytokine signalling pathways (PO00031) were modulated in the infant, young and pubertal groups (78, 82 and 103 genes, respectively) (Fig. 2). Nanoparticle tracking analysis (NTA) revealed that the protein/particle ratio and the production of MSC-derived EVs decreased with increasing donor age (40 ± 2%) (Fig. 3A); however, MSC-derived-EVs production increased with donor age (26 ± 1%) (Fig. 3B). The size of the extracellular vesicles was 160 ± 18 nm, which did not differ significantly among the groups (Fig. 3C). MSC-derived EVs were visualized by electron microscopy as small vesicles, typically 40–80 nm in diameter (Fig. 3D). Flow cytometry analysis of EVs attached to anti-CD63 beads revealed that 32 ± 3% were at least positive for CD63 (an exosome membrane marker protein) at a 10 μM concentration (Fig. 3E).

**Figure 4:**
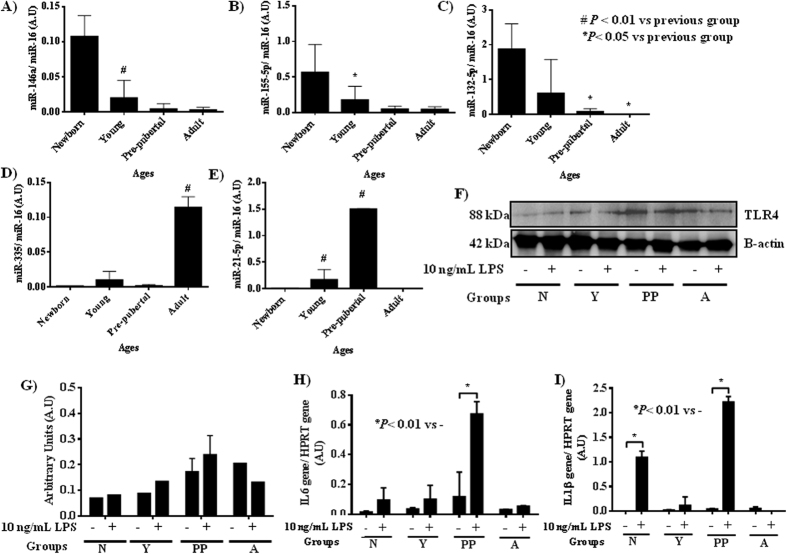
Age-related study of pro-inflammatory factors in mesenchymal stem cell-derived extracellular vesicles. (**A**) miR-146a, (**B**) miR-155, (**C**) miR-132, (**D**) miR-335 and (**E**) miR-21 expression using real-time reverse transcriptase PCR (qRT-PCR) analysis normalized by expression of the housekeeping miR-16. (**F**) Western blot analysis of TLR4 at different ages of MSC groups treated with LPS. *β*-actin was used as the housekeeping baseline. The gels were run under the same experimental conditions. (**G**) Densitometry analysis of western of TLR4 normalized with respect to b-actin. (**H**) *IL-6* gene expression at different ages of MSC groups treated with LPS, using real-time reverse transcriptase PCR (qRT-PCR) analysis normalized by expression of HPRT. (**I**) *IL-1β* gene expression at different ages of MSC groups treated with LPS, using real-time reverse transcriptase PCR (qRT-PCR) analysis normalized by expression of HPRT. N = newborn; Y = young; PP = pre-pubertal and A = adult.

**Figure 5:**
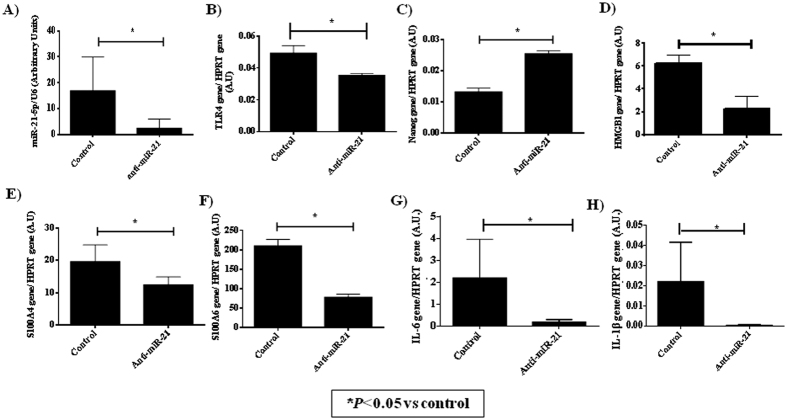
Effect of miR-21-5p on DAMPS and pro-inflammatory factors in mesenchymal stem cells from the pre-pubertal group. (**A**) miR-21-5p expression (**B**) TLR4 gene expression (**C**) Nanog gene expression (**D**) HMGB1 gene expression (**E**) S100A4 gene expression (**F**) S100A6 gene expression (**G**) *IL-6* gene expression (**H**) *IL-1β* gene expression using real-time reverse transcriptase PCR (qRT-PCR) analysis normalized by expression of miR-16 and HPRT.

The qRT-PCR analysis of miRNAs associated with TLR4 (miR-146a; miR-155; miR-132; miR-21 and miR-335), which are also involved in the immunosenescence process, revealed that the expression of miR-146a, miR-155 and miR-132 decreased by 93 ± 3% with increasing donor age. However, the adult group presented the highest statistically significant expression of miR-335 (*P* < 0.01), and the pre-pubertal group presented the highest statistically significant expression of miR-21 (*P* < 0.01) with respect to the other groups (Fig. 4A–D). The TLR4 protein concentration by Western analysis was verified after 4 hours of 10 ng/mL lipopolysaccharide (LPS) treatment (Fig. 4F,G) and a significant increase in the response against LPS was observed in the pre-pubertal group. These data were corroborated by RT-PCR where the highest statistically significant expression of IL-6 and IL-1β (Fig. 4H,I) were observed in the pre-pubertal group in the response against LPS. To explain these results, MSCs from the pre-pubertal group were transiently transfected with miRVana miR-21-5p, and its expression was verified by qRT-PCR (Fig. 5A). The transfected cells expressed statistically significantly less (*P* < 0.05) miR-21 than the same cells transfected with a control mimic miRNA (control). The qRT-PCR analysis of DAMPS associated with TLR4 indicated that miR-21 inhibition promotes a statistically significant decrease in S100A4, S100A6 and HMGB1 with respect to MSC control (Fig. 5C,D,E). *TLR4* gene expression verified by qRT-PCR was statistically significantly lower (*P* < 0.05) than that in the control (Fig. 5B) and *Nanog* gene expression verified by qRT-PCR was statistically significantly higher (*P* < 0.05) than that in the control (Fig. 5C).

**Figure 6:**
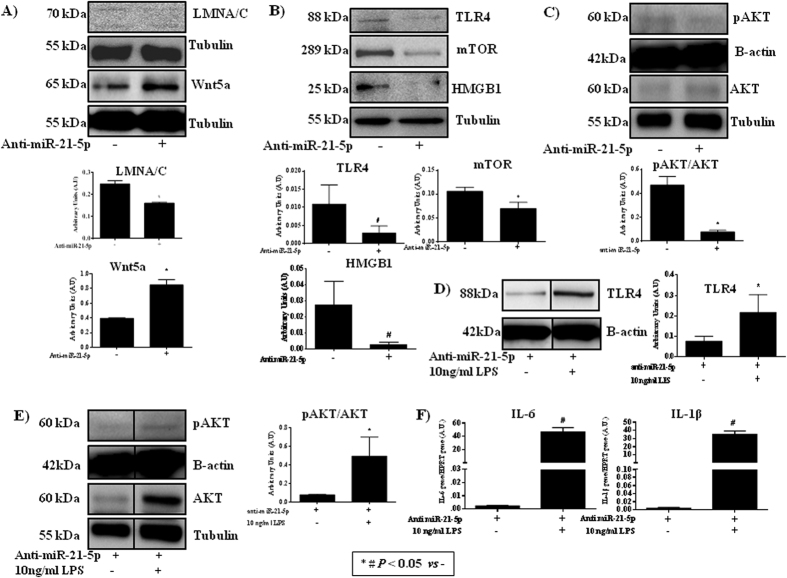
Effect of miR-21-5p on senescence and immune response in mesenchymal stem cells from the pre-pubertal group. (**A**) Western blot analysis of LMNA/C and Wnt5a in the pre-pubertal MSC group with or without inhibition of miR-21 and their densitometry analysis normalized with respect to tubulin. (**B**) Western blot analysis of TLR4, mTOR and HMGB1 in the pre-pubertal MSC group with or without inhibition of miR-21 and their densitometry analysis normalized with respect to tubulin. (**C**) Western blot analysis of the AKT pathway in the pre-pubertal MSC group with or without inhibition of miR-21 and their densitometry analysis normalized with respect to tubulin or *β*-actin. (**D**) Western blot analysis of the TLR4 in the pre-pubertal MSC group miR-21-5p inhibited without or with LPS treatment and their densitometry analysis normalized with respect to *β*-actin. (**E**) Western blot analysis of the AKT pathway in the pre-pubertal MSC group miR-21-5p inhibited without or with LPS treatment and their densitometry analysis normalized with respect to tubulin. The gels were run under the same experimental conditions. Full-length gels were presented in Supplementary Information. (**F**) IL-6 and IL-1*β* gene expressions using real-time reverse transcriptase PCR (qRT-PCR) analysis normalized by expression of HPRT in the pre-pubertal MSC group miR-21-5p inhibited without or with LPS treatment.

Western analysis of proteins involved in the immune response in miR-21-inhibited pre-pubertal MSCs revealed that LMNA/C, TLR4, mTOR and pAKT were statistically significant downregulated (*P* < 0.05) with respect to that in control cells (Fig. 6A–C); HMGB1 was also downregulated in the miR-21-inhibited cells. On the other hand, Wnt5a and AKT were statistically significant (*P* < 0.05) upregulated with respect to the control cells (Fig. 6A,C). miR-21 inhibition did not affect the immune response prior to LPS treatment since TLR4 through pAKT/AKT was statistically significant (P < 0.05) upregulated (Fig. 6D,E) neither through pro-inflammatory factor expressions, *IL-6* and *IL-1β*, were highest statistically significant upregulated (Fig. 6F).

## Discussion

The use of MSCs has been adopted in cell-based therapy due to their multipotency, their low expression of co-stimulatory molecules and their immunosuppressive properties^19^. Although EVs have long been considered cellular artefacts or dust, recent progress in this area indicates that EVs provide intercellular information; they are extracellular organelles that have multifaceted physiological and pathological functions in intercellular communication as well as inter-species and inter-kingdom communication^20^. Martins *et al*.^21^ reported that EVs derived from human bone marrow MSCs had a regenerative potential that had been increasingly recognized. MSC populations from different age groups were characterized by flow cytometry to determine the percentage of cells positive for MSC markers CD29 and CD90 and that were negative for hematopoietic markers (CD34, CD45) (Fig. 1A and Supplementary Information). We did not observe statistically significant differences between the MSC markers in the different MSC age groups studied (data not shown). These results were coincident with the results published by Jin *et al*.^22^,^23^,^24^ indicating that MSCs have similar levels of surface antigen expression including MSCs from different tissues. Even the MSC markers were as abundant as those published by Harting *et al*.^25^.

RNA-Seq analysis is an adequate technique to study gene expression modulation in complex systems^26^,^27^. Our results from RNA-Seq analysis allowed the identification of 9,628 genes that were statistically significant modulated between age groups (Fig. 1B). Our study represents a step further from a previous iTRAQ-based study^23^ where 210 differentially expressed proteins were detected. We used the R/Bioconductor package RamiGO which is an R interface to AmiGO that enables visualization of Gene Ontology (GO) trees^16^. RamiGO provides easy customization of annotation, highlighting of specific GO terms, using terms by P-value. We showed RamiGO functionalities in a genome-wide gene set analysis of genes differentially expressed comparing the six chronologically different age groups from bone marrow-derived MSCs. The genes were grouped into huge metabolic pathways common in all age groups (Fig. 1C). Therefore, our next step focussed on inflammation mediated by chemokine and cytokine signalling pathways (PO00031) and genes that were statistically significant modulated in infant, young and pubertal groups (Fig. 2).

The bioinformatics platform ExoCarta^28^ was used to identify 55 exosome markers in inflammation mediated by chemokine and cytokine signalling pathways. These exosome markers were in concordance with our previous iTRAQ results based on quantitative proteomics^23^. Open-source software for target predictions miRanda and TargetScan were used to identify miRNAs involved with the 55 exosome markers involved in inflammation mediated by chemokine and cytokine signalling pathways. Therefore, we carried out a functional study of miRNA expression involved in inflammation mediated by chemokine and cytokine signalling pathways in infant, young and pubertal groups and their EVs.

EV size was determined by NTA, which calculates the size from the total concentration of the vesicles in solution. We followed the technique used by Gercel-Taylor *et al*.^29^ who reported their optimized method to measure the size distribution of cell-derived vesicles comparable to other analysis instrumentation. We found an increase in the production of MSC-derived EVs from the adult group with respect to the others (Fig. 3A). A rational explanation for this fact is that calcium levels play a role in plasma membrane fusion events involved in adipose accumulation in bone marrow stromal cells with age^30^. We previously observed statistically significant increases in the levels of calcium/calmodulin-dependent protein kinase type II, caldesmon, calponin-1, calponin-3 and calreticulin in the adult group with respect to the others by quantitative proteomics (iTRAQ) analysis^22^. In our present study, MSC-derived EVs have a size in the range of diameter published by Vallabhaneni *et al*.^31^ (Fig. 3A–C) with no significant differences between age groups. These data were validated by electronic microscopy (Fig. 3D) and characterized by flow cytometry, and there were more than 32% positive for CD63, a tetraspanin mainly associated with membranes of intracellular vesicles that it is considered an exosome marker by the International Society for Extracellular Vesicles (ISEV)^32^,^33^ (Fig. 3E).

The acquired immune system shows a functional decline in ability to respond to new pathogens during ageing, whereas serum levels of inflammatory cytokines are increased with age^34^. Inflammaging is a new term coined by Olivieri *et al*.^35^ to name those processes associated with age and their relationship with a loss of expression of the TLR family, a process which could contribute to such inflammation imbalance. There is controversy over the role of TLR4 in pro-inflammatory and differentiation capacities from MSCs^36^ and further research will provide helpful tools for regenerative medicine. We analysed various miRNAs associated with TLR4 contained in MSC-derived EVs. miR-146a is one of the key TLR-induced miRNAs, inhibiting the TLR-signalling pathway by targeting IRAK1 kinase and TRAF6 ligase. miR-132 is a target of IL1R associated kinase IRAK 4, a regulator of the production of inflammatory cytokine^36^. miR-155 is induced via TLR in macrophages and exerts a profound effect on the activity of immune cells^37^,^38^. In our model, we observed a decrease in all of these miRNAs contained in EVs with increasing donor age (Fig. 4A–C) suggesting an association among the decrease of immunologically active EVs and the loss of capacity in activating the immune system through the induction of anti-inflammatory cytokines and T cells. MSC-derived EVs from the adult group contained the highest level of miR-335 (Fig. 4D); this could be associated with cell senescence and loss of therapeutic capacity and linked to the reduced capacity to activate protein kinase D1 (PRKD1), which in turn reduces the activity of the AP-1 transcription factor^39^,^40^. miR-21 negatively regulates LPS-induced lipid accumulation and inflammatory response in macrophages by the TLR4-NF-kB pathway^41^ which is involved in human MSCs during differentiation by regulating SPRY229. We identified the highest level of miR-21 in MSC-derived EVs from the pre-pubertal group and the lowest level in the adult group (Fig. 4E). Therefore, TLR4 could be a target to understand the role of miR-21 in the differentiation of pro-inflammatory capacity depending on donor age.

To clarify the role of miR-21, MSCs from the pre-pubertal group were transiently transfected with miRVana miR-21-5p, and its expression was verified by qRT-PCR (Fig. 5A). The transfected cells expressed statistically significantly lower levels of miR-21 (P < 0.05) than the same cells transfected with a mimic miRNA used as control. qRT-PCR analysis of DAMPS associated with TLR4 indicated that miR-21 inhibition produced a statistically significant decrease in S100A4, S100A6 and HMGB1 with respect to the MSC control (Fig. 5C,D,E). Afterwards expression of IL-6 and IL-1β, associated to TLR4 pathway, were statistically significant decreased produced by miR-21 inhibition (Fig. 5G,H). EV isolation from the cells was not possible due to the nature of transient transfections. BM-MSCs from the adult group have less therapeutic capacity due to TLR4-mediated regulation of bone marrow MSC proliferation and osteogenic differentiation through Wnt3a and Wnt5a signalling^42^. Conversely, TLR4 activation in MSCs from the umbilical cord increased this differentiation to a certain extent^43^. Our results indicated that TLR4 tend to increase with age and that the treatment with LPS did not affect to their immunological response, at short incubation times such as 4 hours (Fig. 4F,G,H,I).

Wnt5a expression augments through TLR4 in response to inflammatory mediators, such as LPS, in several stem cell types and regulated cytokine and chemokine production^44^,^45^. Our results indicate that the inhibition of miR-21 produced an over-expression of Wnt5a accompanied by a decrease of the LMNA/C senescence marker (Fig. 6A), suggesting a role in self-renewal and pluripotent capacities of the MSCs. The increase of Nanog detected by RT-PCR (Fig. 5C) strongly supports this supposition. The inhibition of miR-21 disrupts TLR4 through the AKT/mTOR pathway and immune response because mTOR and pAKT were downregulated (Fig. 6B,C) as well as *IL-6* and *IL-1β* gene expressions (Fig. 5G,H). Similar results were observed by Gharibi *et al*.^46^. These authors proposed that inhibition of the AKT/mTOR pathway affected TLR4. The immunological response of the pre-pubertal MSC group increased statistically significant when miR-21 was inhibited with respect to the pre-pubertal MSC control (Fig. 6D). On the other hand, TLR4, pAKT/AKT, *IL-6* and *IL-1β* increased their expression in the miR-21-inhibited pre-pubertal MSC group after treatment with LPS. This result indicates that miR-21 affected DAMPS and TLR4 levels but not their immunological response of the MSCs to outside agents such as LPS (Fig. 6E,F) thus, suggesting that miR-21 could be a regulator of TLR4 signalling^46^,^47^,^48^.

## Conclusion

Our results provide insight into the mechanism involved in MSC ageing and suggest possible interventions in miRNAs to maintain quiescence and the function of MSCs and their derived extracellular vesicles prior to *in vivo* transplantation or as pharmacological agents in disease.

## Additional Information

**How to cite this article:** Fafián-Labora, J. *et al*. Effect of age on pro-inflammatory miRNAs contained in mesenchymal stem cells-derived extracellular vesicles. *Sci. Rep.* **7**, 43923; doi: 10.1038/srep43923 (2017).

**Publisher's note:** Springer Nature remains neutral with regard to jurisdictional claims in published maps and institutional affiliations.

## Supplementary Material

Supplementary Information

## Figures and Tables

**Table 1:** Specific primers for real-time reverse transcriptase-polymerase chain reaction (qRT-PCR) amplification.

Target	mRNA ID	Forward (5′-3′)	Reverse (5′-3′)
HMGB1	NM_012963.2	CCGGATGCTTCTGTCAACTT	TTGATTTTTGGGCGGTACTC
S100A4	NM_012618.2	AGCTACTGACCAGGGAGCTG	CTGGAATGCAGCTTCGTCT
S100A6	NM_053485.2	TGATCCAGAAGGAGCTCACC	AGATCATCCATCAGCCTTGC
NANOG	NM_005103.4	ATGCCTCACACGGAGACTGT	AAGTGGGTTGTTTGCCTTTG
TLR4	NM_019178.1	GCAGAAAATGCCAGGATGATG	AAGTACCTCTATGCAGGGATTAG
IL-6	NM_012589.2	CCTTTCAGGAACAGCTATGAA	ACAACATCAGTCCCAAGAAGG
IL-1β	NM_031512.2	TGTGATGAAAGACGGCACAC	CTTCTTCTTTGGGTATTGTTTGG
