# Corrigendum: Effect of age on pro-inflammatory miRNAs contained in mesenchymal stem cell-derived extracellular vesicles

**DOI:** 10.1038/srep46850

**Published:** 2017-06-15

**Authors:** J. Fafián-Labora, I. Lesende-Rodriguez, P. Fernández-Pernas, S. Sangiao-Alvarellos, L. Monserrat, O. J. Arntz, F. A. J. van de Loo, J. Mateos, M. C. Arufe

Scientific Reports 7: Article number: 43923; 10.1038/srep43923 published online: 03 06 2017; updated: 06 15 2017.

The original version of this Article contained a typographical error in the spelling of the author F. A. J. van de Loo, which was incorrectly given as F. J. Van de Loo.

In addition, there were errors in Affiliation 4 which was incorrectly listed as ‘Experimental Rheumatology, Radboudumc University Medical Center, Huispost 272, route 272, Postbus 9101, 6500 HB Nijmegen, The Netherlands’. The correct affiliation is listed below:

Experimental Rheumatology, Department of Rheumatology, Radboud university medical center, Nijmegen 6525 GA, The Netherlands.

These errors have now been corrected in both the PDF and HTML versions of the Article.

