# Supplementary material for: Effect of age on pro-inflammatory miRNAs contained in mesenchymal stem cell-derived extracellular vesicles
Source: Sci Rep. 2017 Mar 6;7:43923. doi: 10.1038/srep43923 (PMC5338265; doi:10.1038/srep43923)
Supplement: Supplementary Information [file srep43923-s1.pdf]

Effect of age on pro-inflammatory miRNAs contained in mesenchymal stem cells-derived extracellular vesicles

J. Fafián-Labora[1], I. Lesende-Rodriguez[1], P. Fernández-Pernas[1], S. Sangiao-Alvarellos[2], L. Monserrat[3], O. J. Arntz[4], F. J. Van de Loo[4], J. Mateos[1#], *MC. Arufe[1]

Supplementary Information Fig. 1A

Representative flow graph from newborn group characterized by flow cytometry assay

Supplementary Information Fig. 6

Full-length gels used to make the figure 6. A) Western blots of LMNA/C, Wnt5a and tubulin in pre-pubertal MSCs group with or without inhibition of miR-21. B) Western blots of TLR4, mTOR, HMGB1 and tubulin in pre-pubertal MSCs group with or without inhibition of miR-21. C) Western blots of AKT, tubulin, pAKT and B-actin in pre-pubertal MSCs group with or without inhibition of miR-21. D) Western blot of TLR4 and B-actin in pre-pubertal MSCs group miR-21-5p inhibited with or without LPS treatment.

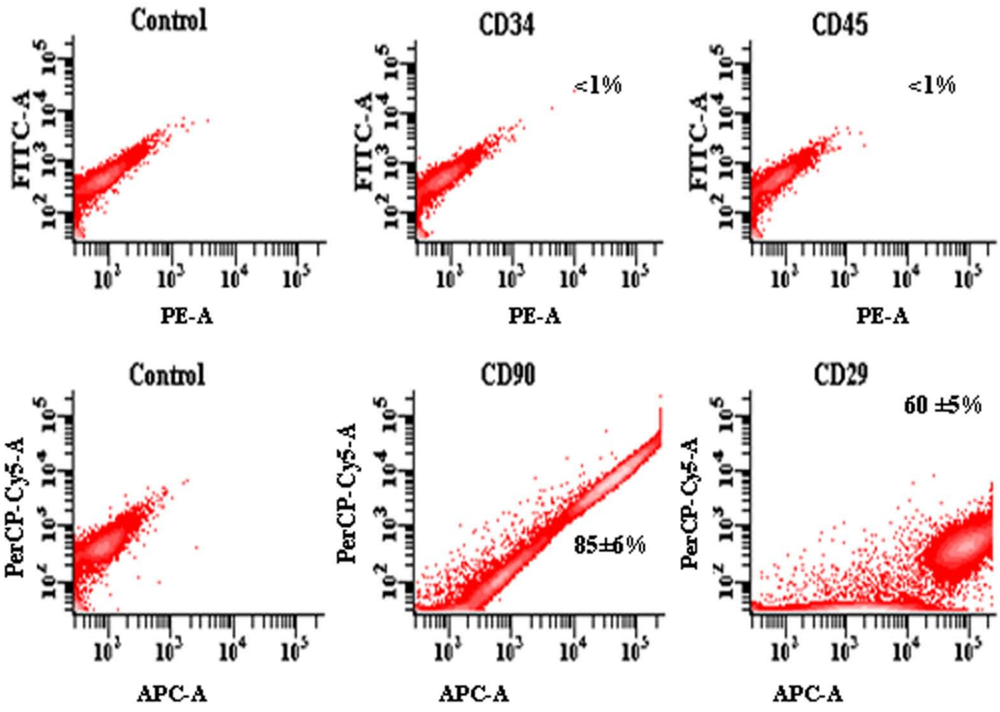

Supplementary Information Fig. 1A

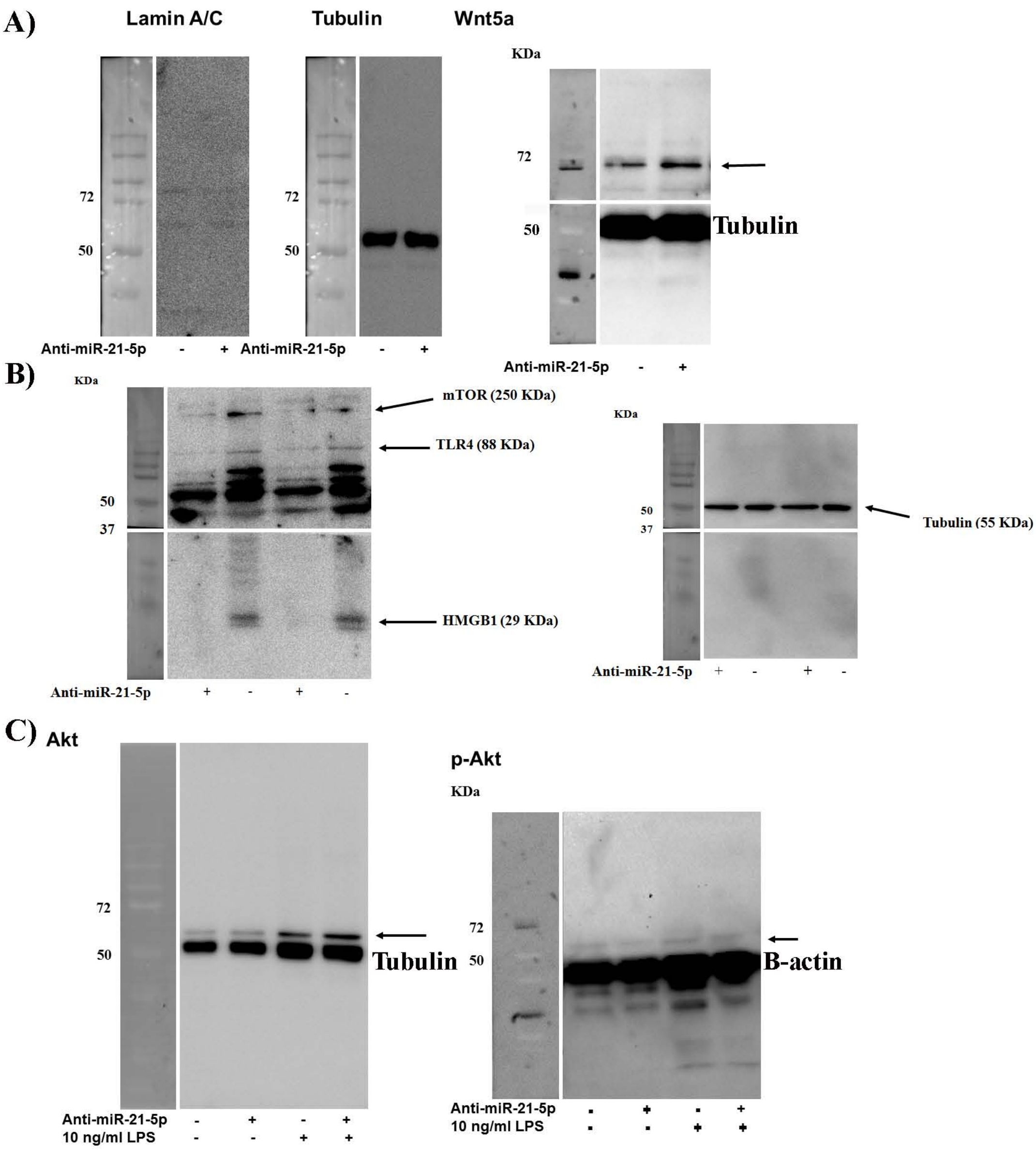

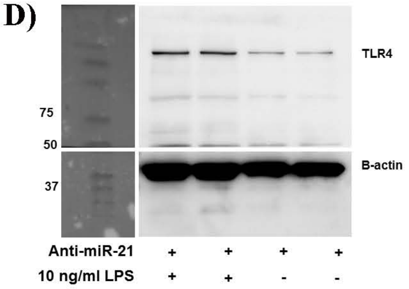

Supplementary information Fig. 6
